# Uranium–Lead Systematics of Lunar Basaltic Meteorite Northwest Africa 2977

**DOI:** 10.5702/massspectrometry.A0115

**Published:** 2023-02-10

**Authors:** Narumi Moromoto, Yosuke Kawai, Kentaro Terada, Masaaki Miyahara, Naoto Takahata, Yuji Sano, Naoko Fujikawa, Mahesh Anand

**Affiliations:** 1 Department of Earth and Space Science, Graduate School of Science, Osaka University, 1–1 Machikaneyama, Toyonaka, Osaka 560–0043, Japan; 2 Forefront Research Center, Graduate School of Science, Osaka University, 1–1 Machikaneyama, Toyonaka, Osaka 560–0043, Japan; 3 Graduate School of Advanced Science and Engineering, Hiroshima University, Higashi-Hiroshima, Hiroshima 739–8526, Japan; 4 Atmosphere and Ocean Research Institute, The University of Tokyo, Kashiwa, Chiba 277–8564, Japan; 5 Center for Advanced Marine Core Research, Kochi University, Nankoku, Kochi 783–8502, Japan; 6 School of Physical Sciences, The Open University, Milton Keynes MK7 6AA, UK; 7 Department of Earth Sciences, The Natural History Museum, London SW7 5BD, UK

**Keywords:** secondary ionization mass spectrometry, *in-situ* isotopic analysis, lunar meteorite

## Abstract

Northwest Africa (NWA) 2977 is a lunar basaltic meteorite that was found in 2005 and has been classified as an olivine cumulate gabbro. This meteorite contains a shock melt vein (SMV) induced by an intense shock event. We report herein on an *in-situ* analysis of phosphates in the host gabbro and the shock vein for the U–Pb dating of NWA 2977 using an ion microprobe, NanoSIMS. The majority of the analyzed phosphates, in both the SMV and host-rock, lie on a linear regression in ^238^U/^206^Pb–^207^Pb/^206^Pb–^204^Pb/^206^Pb three-dimensional space, indicating a total Pb/U isochron age of 3.15±0.12 Ga (95% confidence level), which is consistent ages determined in previous isotopic studies of NWA 2977 (Sm–Nd age of 3.10±0.05 Ga, Rb–Sr age of 3.29±0.11 Ga, and Pb–Pb baddeleyite age of 3.12±0.01 Ga), and identical to the age of the U–Pb phosphate in a paired meteorite NWA 773, 3.09±0.20 Ga, derived from our dataset. There was no clear difference in the formation age between the phosphates found in the SMV and host-rock, although the shape and size of the grains and the Raman spectra show the evidence of intense shock metamorphism. Based on these findings, the cooling rate of the phosphate was very rapid, constrained to be larger than 140 K/s.

## INTRODUCTION

The Apollo and Luna missions, which returned approximately 380 kg of lunar rock and soil samples, have formed the basis for many studies regarding crustal evolution and magmatism on the Moon. However, these samples were collected at broadly equatorial regions of the nearside of the Moon.^1,2)^ Remote sensing data show that the landing sites are in or near to the geochemically anomalous region of the Moon known as the Procellarum KREEP Terrane.^3)^ Thus, these samples cannot be considered to be representative of the majority of the material on the surface of the Moon. However, in the past four decades, many lunar meteorites have been discovered in hot deserts and in Antarctica, potentially providing new insights into unexplored areas of the Moon, beyond the nearside equatorial region (*e.g.*, ref. 4,5).

In this paper, we report on an *in-situ* U–Pb isotopic analysis of phosphate grains in lunar meteorite Northwest Africa 2977 (hereafter, NWA 2977) that was found in Morocco or Algeria in 2005.^6)^ NWA 2977 consists mainly of olivine, pigeonite, augite, and plagioclase with accessory minerals including K-feldspar, chromite, ilmenite, phosphate, baddeleyite, troilite and Fe–Ni metal.^7–9)^ Based on this mineral composition, NWA 2977 was classified as an olivine cumulate gabbro (OC), suggesting that the rock originated from a mare region of the Moon. The investigated sample contains a shock-induced melt vein structure 1.5 cm in length cutting across the host-rock.

Several radiometric ages of NWA 2977 have been reported in previous studies. For example, Burgess *et al.* reported a whole-rock age of 2.77±0.04 Ga using the Ar–Ar method^10)^ (unless otherwise noted, uncertainties are all reported at the 95% confidence level in the following text). In contrast, Sm–Nd and Rb–Sr isotopic systems of whole-rocks and minerals yielded older ages of 3.10±0.05 Ga and 3.29±0.11 Ga, respectively,^11)^ which are consistent with the Pb–Pb age of 3.12±0.01 Ga obtained from *in-situ* isotopic analyses of baddeleyite.^8)^ These reported ages are relatively young compared to the majority of mare basalt samples obtained in the Apollo and Luna missions, with ages ranging from 4.3 to 3.2 Ga.^12,13)^

For a better understanding of the thermal history recorded in NWA 2977, we conducted *in-situ* U–Pb isotopic measurements of phosphate grains in the host-rock and shock melt vein (hereafter SMV) using an ion microprobe. Phosphates are important carriers of U in lunar basalt and are resistant to thermal disturbance in the U–Pb system. The U–Pb dating method has an advantage over other dating methods because even in the case where some disturbance has affected the U–Pb systematics, both the primary crystallization age and the age of the secondary event can be determined by the assessment of both the ^238^U and ^235^U decay series unless it was completely reset. In a previous study, we demonstrated the robustness of our U–Pb *in-situ* dating technique for dating crystallization and alteration ages of phosphates in lunar basaltic rocks^14,15)^ and martian meteorites.^16,17)^ To confirm the obtained result, the age determined for the phosphates in NWA 2977 was compared with similar analyses of phosphates in NWA 773, which is considered to be a paired meteorite.^7–9)^

## SAMPLE AND ANALYTICAL METHODS

We embedded a 1 cm^2^ sized fragment of NWA 2977 in an epoxy resin (Araldite 502) and polished it. To identify the location and mineralogy of the phosphate phases, back-scattered electron (BSE) images of the polished sample were obtained, followed by measurement of elemental compositions, using a JEOL JSM-6010LA scanning electron microscope (SEM) with a Thermo Scientific™ NORAN™ System 7 energy dispersive X-ray spectrometer (EDS) system at an accelerating voltage of 20 kV. To prevent charging during the SEM-EDS analysis, the sample surface was carbon-coated.

We then conducted the isotope analysis with the NanoSIMS at the Atmosphere and Ocean Research Institute (AORI), The University of Tokyo.^18,19)^ Before the measurement, the carbon-coating was removed and the sample surface was gold-coated to avoid charging by primary ion beam irradiation. To reduce interference of any protonated molecules attributed to water absorbed on the surface of the mount, the sample was evacuated overnight with some heat provided by a lamp in the sample lock of the NanoSIMS. Since the size of the phosphates in the SMV was less than 10 μm in diameter, the primary O^−^ ion beam of 0.3 nA was focused to sputter an area 4 μm in diameter on the phosphates. Before the actual measurement, the primary ion beam was rastered on the sample surface for 5 min to remove the gold coating and reduce the contribution of surface contaminant Pb to the analysis.

Generated positive ions were extracted with an acceleration voltage of 8 kV and introduced into a mass analyzer with a Mattauch–Herzog geometry which enables multiple secondary ions to be simultaneously detected under a static magnetic field. For ^238^U–^206^Pb dating, the secondary ions, ^204^Pb^+^, ^206^Pb^+^, ^238^UO^+^, and ^238^UO_2_^+^ together with phosphate matrix ion peaks of ^31^P^+^ and ^43^Ca^+^ were collected simultaneously with the multi-collector system. A mass resolution of 4100 at a 1% peak height was set to separate ^206^Pb^+^ and ^143^Nd^31^PO_2_^+^ (the major isobaric molecule in apatite (Ca_5_(PO_4_)_3_(F, Cl, OH)) matrix). One analysis required 10 min to obtain statistically sufficient ion counts.

**Figure 1:**
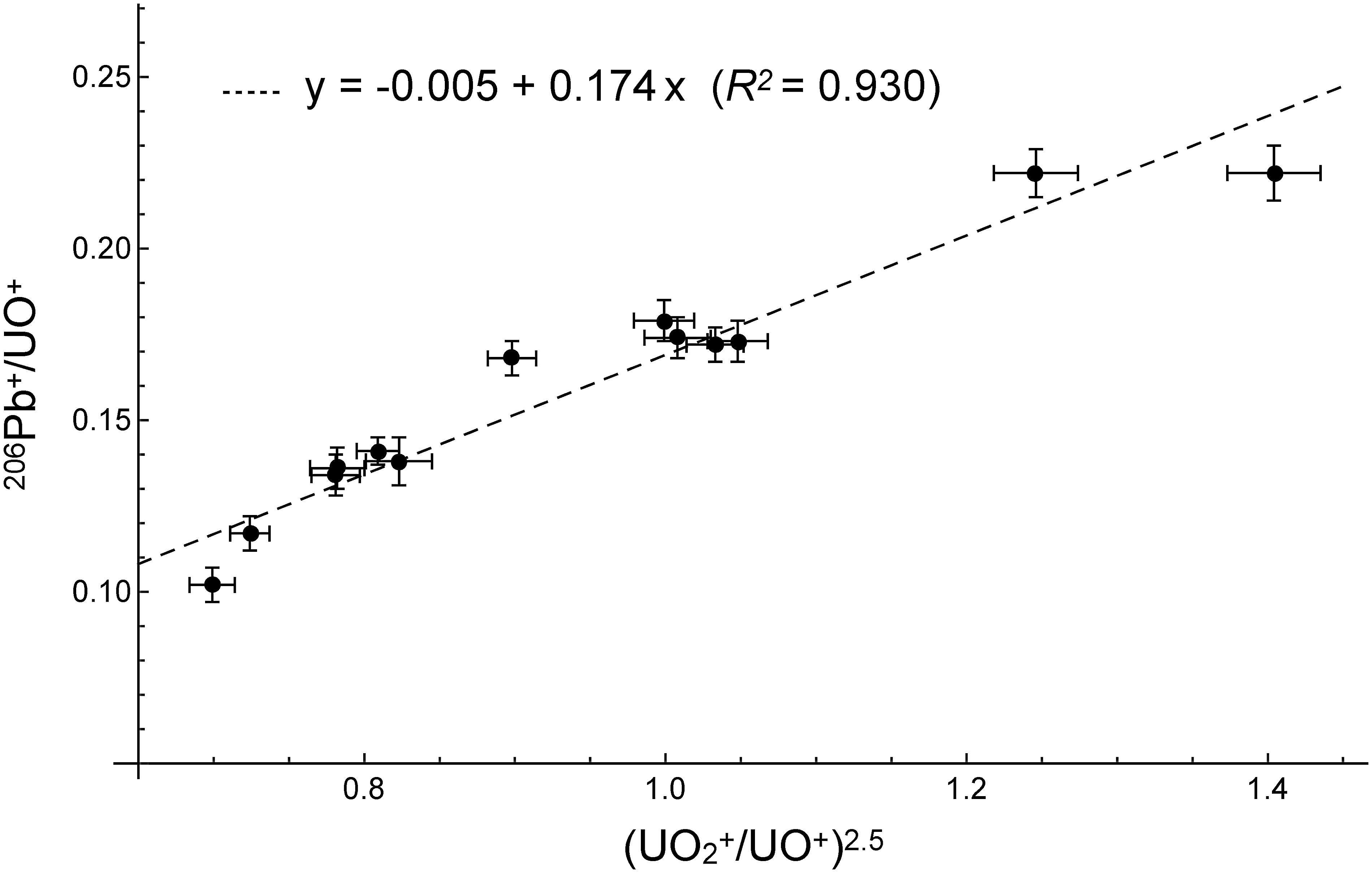
Fig. 1. The relationship between ^206^Pb^+^/^238^UO^+^ and ^238^UO_2_^+^/^238^UO^+^ of the PRAP standard obtained in the one-week analytical session of this study. The slope and intercept of the linear regression line (*R^2^*=0.930) are 0.174 and −0.005, respectively.

For the NanoSIMS analysis, secondary U ions are mainly observed as monoxide and dioxide species, while Pb is emitted almost entirely as Pb^+^.^18)^ Therefore, the ^238^U/^206^Pb ratio was obtained by applying the observed ^206^Pb^+^/^238^UO^+^ and ^238^UO_2_^+^/^238^UO^+^ ratios to the empirical relationship for standard apatite, PRAP derived from an alkaline rock of the Prairie Lake circular complex in the Canadian Shield dated at 1.15±0.02 Ga.^20)^ The relationship between ^206^Pb^+^/^238^UO^+^ and ^238^UO_2_^+^/^238^UO^+^ of the PRAP standard is expressed as 
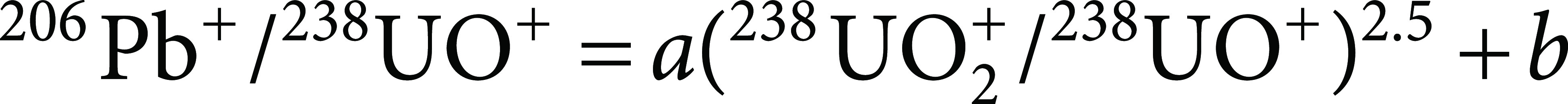
 where *a* and *b* are constants determined by repeated measurements of the PRAP. Figure 1 shows the relationship obtained in the one-week analytical session of this study. The constants *a* and *b* are 0.174 and −0.005, respectively. With this relationship, the following ^238^U/^206^Pb ratio of the sample was obtained:
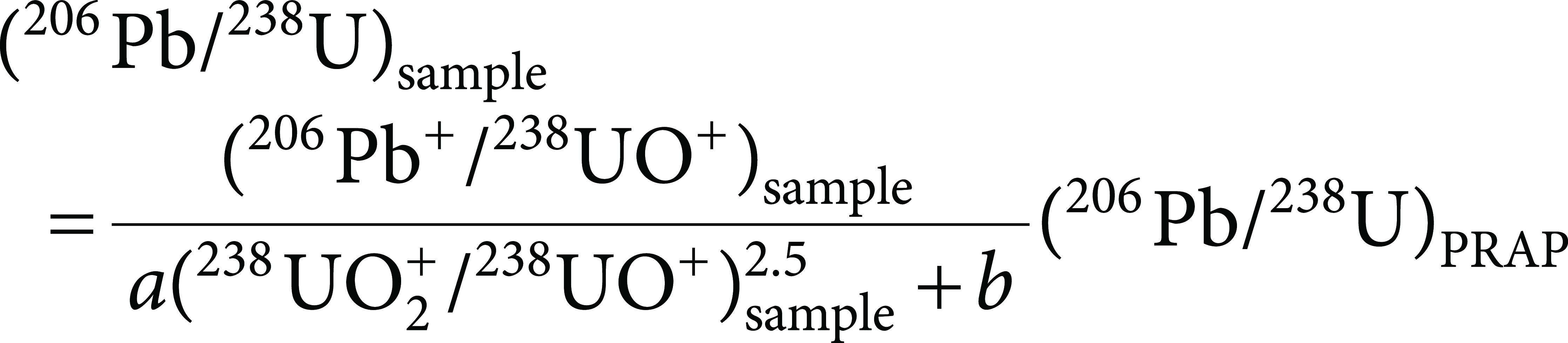
 (^206^Pb/^238^UO)_PRAP_ is determined using ^238^U–^206^Pb age of 1.15±0.02 Ga. More details of the data calibration are presented elsewhere.^19)^

After the ^238^U/^206^Pb measurement, for ^207^Pb–^206^Pb dating, ^204^Pb^+^, ^206^Pb^+^, and ^207^Pb^+^ were collected on the same spots with a single collector by scanning the magnetic field.^18)^ Approximately 1 h was required to attain statistically sufficient counts. In this way, the ^238^U/^206^Pb, ^207^Pb/^206^Pb and ^204^Pb/^206^Pb ratios, which are required to calculate the U–Pb age were obtained.

In this study, for a comparison of U–Pb systematics of NWA 2977 and NWA 773, the data for NWA 773, which has not been reported previously, were used. The polished thin section of the sample was measured with a Sensitive High Resolution Ion Microprobe (SHRIMP) at Hiroshima University. The measurement and data reduction method for the U–Pb systematics of phosphate with SHRIMP is similar to that of NanoSIMS. The phosphates were irradiated with a primary O_2_^−^ ion beam of 1.0 nA with a spot diameter of about 10 μm. Before the analysis, the sample was evacuated overnight in the sample lock, and the primary ion beam was rastered on the sample surface for 3 min to remove any possible remaining contaminants. The positive secondary ions, ^40^Ca_2_^31^PO_3_^+^, ^202^Hg^+^, ^204^Pb^+^, ^206^Pb^+^, ^207^Pb^+^, ^208^Pb^+^, ^238^U^+^, ^232^ThO^+^, and ^238^UO^+^, were detected on a single electron multiplier by cyclically peak-stepping the magnetic field. The mass resolution was set to 5800 at ^208^Pb. The ^238^U/^206^Pb ratio was also obtained from the empirical relationship between ^206^Pb^+^/^238^U^+^ and ^238^UO^+^/^238^U^+^ ratios of the PRAP, instead of ^206^Pb^+^/^238^UO^+^ and ^238^UO_2_^+^/^238^UO^+^. The details were reported in previous studies (*e.g.*, ref. 21, 22).

In order to assess shock metamorphism, Raman spectroscopy of the phosphates was performed using a laser micro-Raman: JASCO NRS-5100 instrument at Tohoku University. A microscope was used to focus the excitation laser beam (the 531.92 nm line of the green laser). The laser power was maintained at less than 7 mW to reduce damage caused by the laser beam. The acquisition time was 30 s. A Raman spectrum was acquired for each phase in the spectral region of 300 to 1200 cm^−1^.

## RESULTS

**Figure 2:**
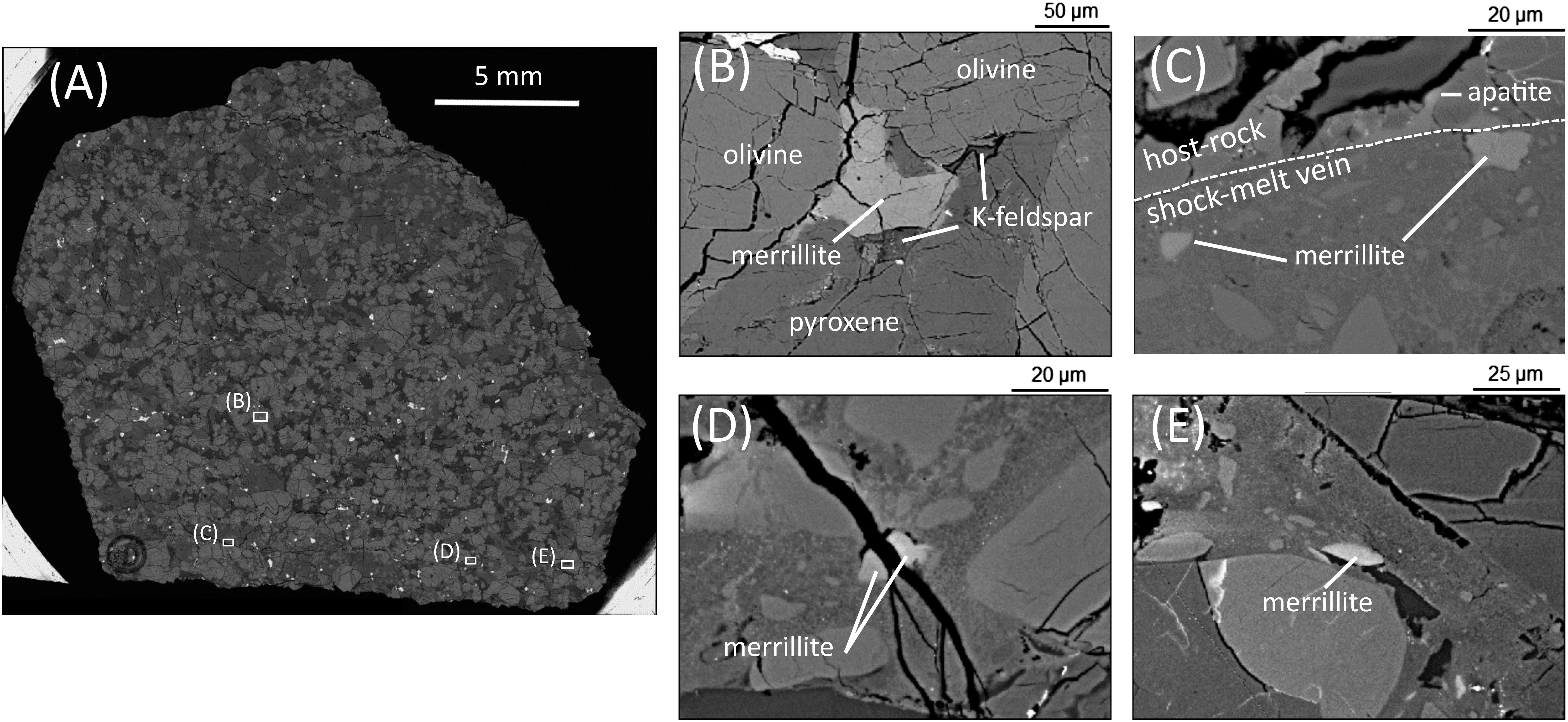
Fig. 2. (A) Back scattered electron (BSE) image of the whole area of the polished NWA 2977 sample. (B) Enlarged BSE image of phosphate grains in the host-rock of NWA 2977. There is a merrillite grain in the center of the image and its size is more than 50 μm. (C)–(E) Enlarged BSE image of the shock melt vein (SMV). (C) The dashed line indicates the boundary between the host-rock and SMV. In the fine-grained matrix of SMV, there are two small grains of phosphate (merrillite) with a rounded shape.

Figure 2A shows the BSE image of the whole area of the polished NWA 2977 sample that was investigated in this study. The major constituents of the host-rock are olivine, pyroxene, and plagioclase as reported in detail in previous studies.^7–9)^ A 20 μm wide SMV cuts through the host-rock. The SMV consists of coarse-grained fragments and a fine-grained matrix which crystallized from a melt of the host-rock material. The sizes of the fragmented minerals entrained in the vein are typically ∼5 μm, smaller than those of the grains in the host-rock (typically tens of micrometers). Two types of phosphate, apatite (Ca_5_(PO_4_)_3_(F, Cl, OH)) and merrillite (Ca_9_NaMg(PO_4_)_7_), were found in NWA 2977, with merrillite being more abundant than apatite. The phosphates occurring in the host-rock had various shapes and sizes (irregular boundaries to adjacent grains and with diameters smaller than 10 μm to larger than 50 μm), as shown in Fig. 2B. In contrast, phosphates in the SMV were relatively rounded and all grain sizes are less than 10 μm in diameter, as shown in Fig. 2C–E.

**Figure 3:**
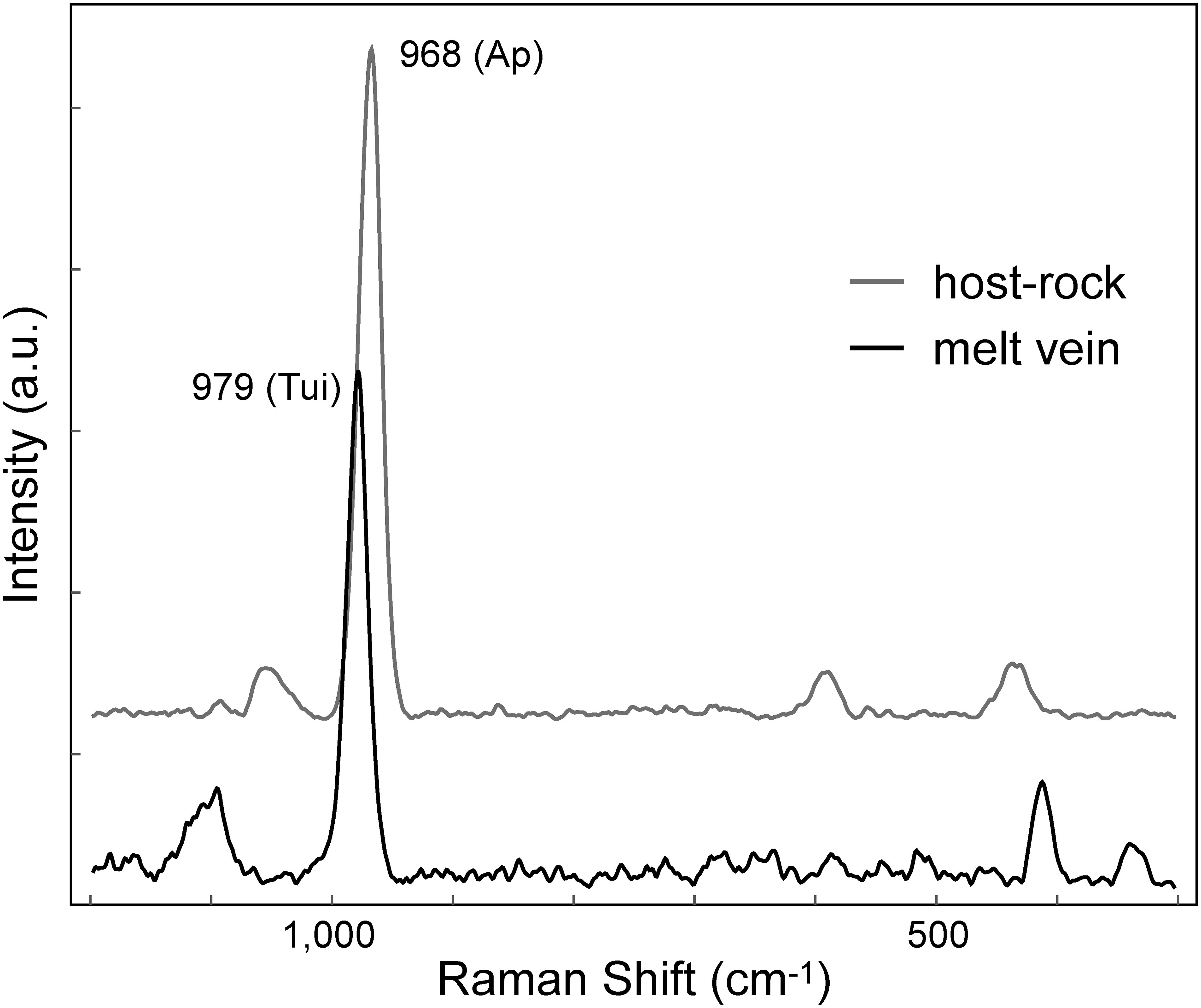
Fig. 3. Representative Raman spectra of apatite (Ap) in the host-rock (gray line) and tuite (Tui) in the SMV (black line) of NWA 2977. The intense peak at around 968 cm^−1^ in the host-rock is shifted to around 979 cm^−1^ in the SMV, showing that the grains in the SMV have been partially transformed into its high-pressure polymorph.

Figure 3 shows representative Raman spectra of the apatite in the host-rock and SMV. The intense peak at around 968 cm^−1^ in the host-rock is shifted to around 979 cm^−1^ in the SMV, showing that the grains in the SMV have been partially transformed into its high-pressure polymorph, tuite.^23,24)^

We carried out *in-situ* U–Pb measurements on 24 phosphate grains using NanoSIMS. Nine data points were excluded from the analysis because the measurements were made on cracks or grain boundaries. A total of 6 merrillite grains in the SMV, and 5 apatite and 4 merrillite grains in the host-rock were analyzed and the obtained ^238^U/^206^Pb, ^207^Pb/^206^Pb, ^204^Pb/^206^Pb ratios are summarized in Table 1. The isotopic data of the phosphates found in two lithologies of NWA 773, olivine cumulate gabbro (OC) and polymict, fragmental regolith breccia (BX),^25)^ are also summarized in Table 1.

**Table 1:** Table 1. The observed ^238^U/^206^Pb–^207^Pb/^206^Pb–^204^Pb/^206^Pb ratios in phosphates and silicates in NWA 2977 and NWA 773. The analytical uncertainties are 1 sigma.

	Name	Mineral	^238^U/^206^Pb	^207^Pb/^206^Pb	^204^Pb/^206^Pb
NWA 2977 (host rock)	A-1	Apatite	2.010±0.372	0.245±0.011	0.00061±0.00330
	A-2-3	Apatite	1.459±0.186	0.240±0.016	0.00020±0.00122
	B-1	Apatite	1.494±0.089	0.255±0.018	0.00297±0.00175
	B-2-1	Merrillite	1.369±0.068	0.239±0.014	0.00125±0.00137
	B-3-1	Merrillite	1.337±0.135	0.235±0.011	0.00081±0.00090
	C#-1-1	Merrillite	1.324±0.083	0.241±0.014	0.00100±0.00083
	D-2-1	Apatite	1.455±0.088	0.243±0.016	0.00102±0.00166
	D-2-2	Merrillite	1.444±0.082	0.252±0.013	0.00932±0.00256
	D-2-3	Apatite	1.454±0.085	0.243±0.012	0.00117±0.00086
NWA 2977 (melt vein)	C-2-2	Merrillite	1.360±0.378	0.230±0.009	0.00033±0.00196
	C-2-3	Merrillite	1.156±0.075	0.238±0.007	0.00028±0.00062
	E-2-1	Merrillite	1.128±0.072	0.241±0.010	0.00049±0.00064
	E-3-1	Merrillite	1.542±0.283	0.246±0.010	0.00069±0.00220
	E-3-2	Merrillite	1.259±0.203	0.246±0.012	0.00015±0.00091
	E-4	Merrillite	1.311±0.170	0.241±0.012	0.00041±0.00089
NWA 773	NWA773-AA1	Apatite	1.053±0.439	0.217±0.032	0.00038±0.00005
	NWA773-AB1	Apatite	1.607±0.280	0.236±0.011	0.00050±0.00011
	NWA773-BA1	Merrillite	1.400±0.167	0.274±0.013	0.00112±0.00011
	NWA773-CA1	Merrillite	1.621±0.125	0.237±0.038	0.00034±0.00003
	NWA773-CB1	Merrillite	1.488±0.229	0.245±0.011	0.00124±0.00009
	NWA773-D-1	Merrillite	1.115±0.174	0.243±0.008	0.00056±0.00008
	NWA773-GA1	Merrillite	1.139±0.983	0.260±0.034	0.00062±0.00012
	NWA773-GB1	Apatite	1.034±0.229	0.250±0.023	0.00024±0.00004
	NWA773-HA1	Merrillite	1.508±0.222	0.254±0.010	0.00072±0.00018
	NWA773-HB1	Merrillite	1.425±0.274	0.228±0.009	0.00028±0.00005
	NWA773-HB2	Merrillite	1.038±0.203	0.242±0.017	0.00006±0.00002
	NWA773-IA1	Merrillite	0.957±0.456	0.251±0.026	0.00054±0.00045
	NWA773-IA2	Merrillite	0.972±0.428	0.252±0.024	0.00062±0.00014
	NWA773-IB1	Merrillite	1.079±2.876	0.245±0.024	0.00054±0.00010
	NWA773-BB1	Merrillite	2.074±0.391	0.255±0.013	0.00075±0.00007

**Figure 4:**
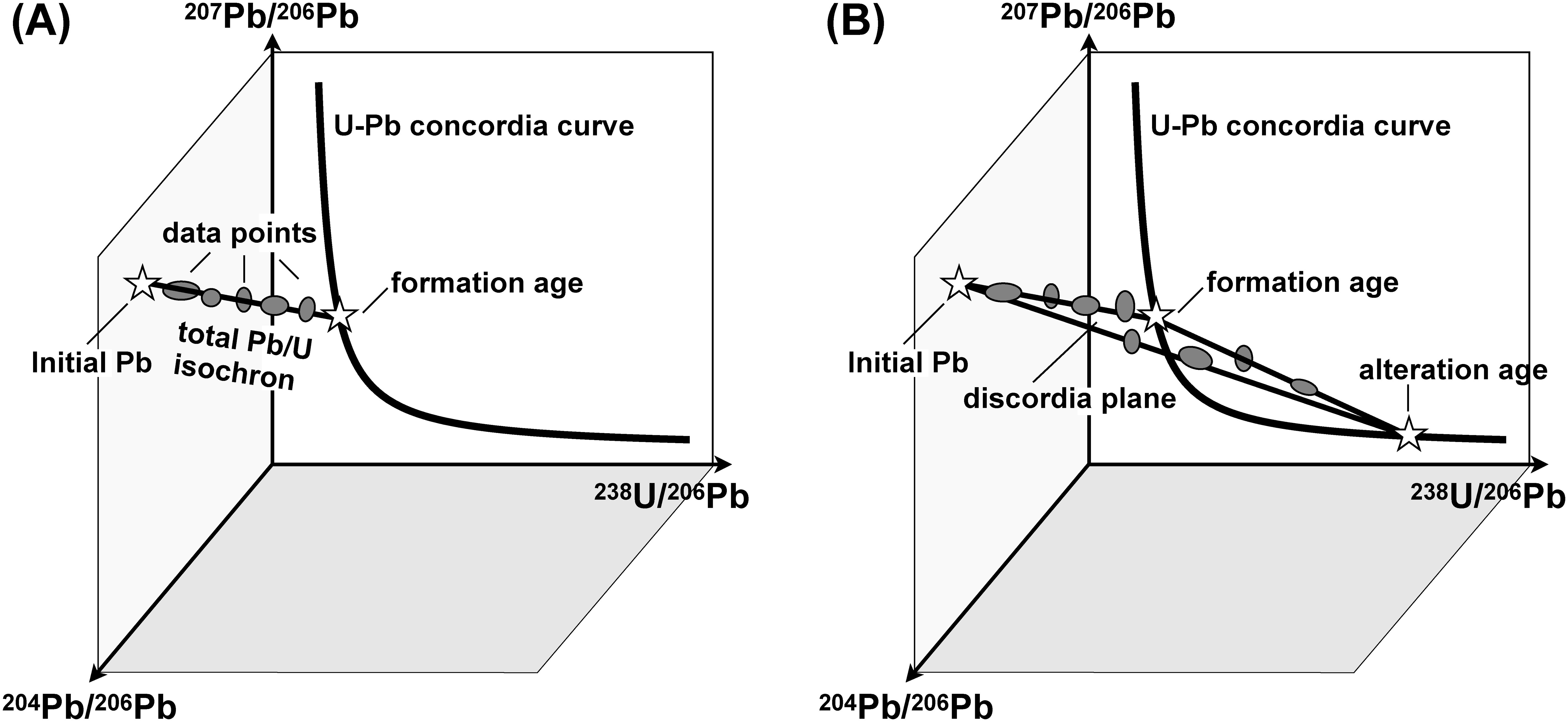
Fig. 4. Schematic diagram of total Pb/U isochron method in the ^238^U/^206^Pb–^207^Pb/^206^Pb–^204^Pb/^206^Pb 3-D space for the concordia case (A) and the discordia case (B). (A) For the concordia case, the formation age is determined as the intersection of a regression line (total Pb/U isochron) with the U–Pb concordia curve on the ^238^U/^206^Pb–^207^Pb/^206^Pb plane. (B) For the discordia case, the upper and lower intercepts of the planar regression (discordia plane) with the concordia curve correspond to the formation age and the alteration age, respectively.

We calculated the total Pb/U isochron age in the ^238^U/^206^Pb–^207^Pb/^206^Pb–^204^Pb/^206^Pb three-dimensional (3-D) space (for details see ref. 26–28). Figure 4A shows a schematic diagram of a 3-D linear regression for the total Pb/U isochron. The isotopic ratios of the samples with an undisturbed U–Pb system that share the same initial Pb isotopic composition lie on a linear regression line in the ^238^U/^206^Pb–^207^Pb/^206^Pb–^204^Pb/^206^Pb space. The formation age of the samples is determined as the intersection of the regression line with the U–Pb concordia curve on the ^238^U/^206^Pb–^207^Pb/^206^Pb plane, and the intersection with the ^207^Pb/^206^Pb–^204^Pb/^206^Pb plane corresponds to the isotopic composition of the initial Pb. The crucial advantages of this method are that it is not necessary to know the initial lead isotopic ratio and that a justifiable age with a smaller uncertainty can be obtained by simultaneously using both ^238^U and ^235^U systematics. Moreover, as described above, if a secondary event has a slight affect on the U–Pb systematics, the observed data are scattered on a plane in 3-D space due to Pb-loss (Fig. 4B). The upper and lower intercepts of the planar regression (discordia plane) with the concordia curve correspond to the formation age and the alteration age, respectively.

**Figure 5:**
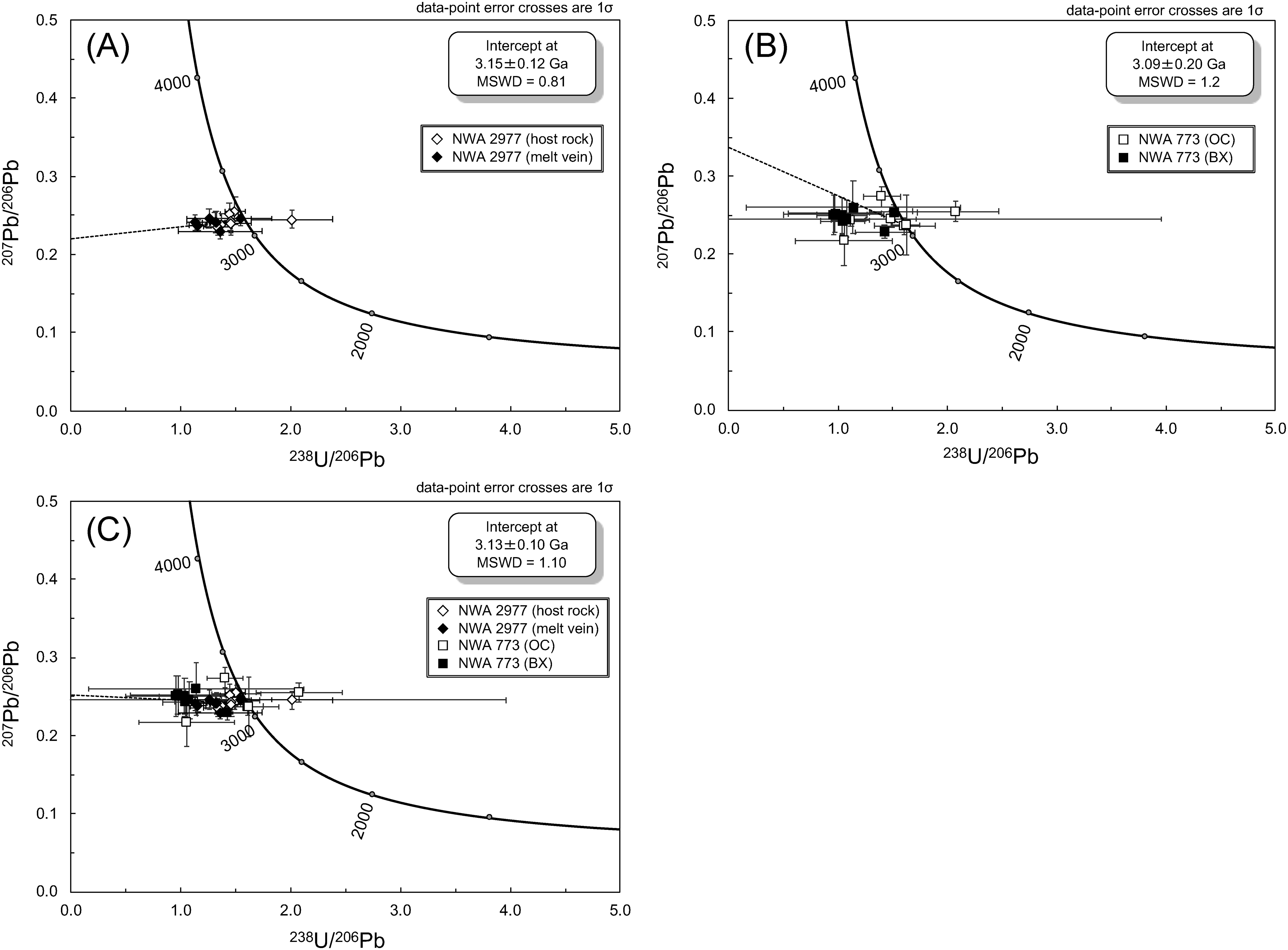
Fig. 5. The results of the linear regressions of phosphates in the ^238^U/^206^Pb–^207^Pb/^206^Pb–^204^Pb/^206^Pb three-dimensional (3-D) space. (A) and (B) shows the result for the phosphates in NWA 2977 and NWA 773, respectively, and (C) the combined results for NWA 2977 and 773. In (A), the phosphates in the SMV and host-rock are indicated as open diamonds and filled diamonds, respectively. In (B), the phosphates in the OC and BX lithologies are indicated as open and filled squares, respectively. The data points and the linear regression line (dashed line) in 3-D space are projected onto the ^238^U/^206^Pb–^207^Pb/^206^Pb plane. The solid line expresses the concordia line. The error bars assigned to the data points are 1 sigma.

Figure 5A and B shows the isotopic ratios and the linear regression obtained for the total Pb/U isochron projected onto the ^238^U/^206^Pb–^207^Pb/^206^Pb plane for NWA 2977 and NWA 773, respectively. It should be noted for NWA 2977 that there is no clear difference in the isotopic compositions between the phosphates found in the SMV and host-rock. For NWA 773, no clear difference was seen in the isotopic ratios of the phosphates in the OC and BX lithologies.

The U–Pb data from 5 apatite and 10 merrillite grains in NWA 2977 are well expressed by a linear regression within analytical uncertainties, indicating that the U–Pb system in these grains has not been significantly disturbed. The intersection with the concordia curve gives an isochron age of 3.15±0.12 Ga for NWA 2977. The data from 3 apatites and 12 merrillites in NWA 773 are also well expressed by a linear regression, giving a total Pb/U isochron age 3.09±0.20 Ga. All obtained ages are in agreement within the analytical uncertainties, supporting the argument for a paired relationship of NWA 2977 and NWA 773.^7–9)^ The combined U–Pb systematics of NWA 2977 and NWA 773 (a total of 8 apatites and 22 merrillites) gives an isochron age of 3.13±0.10 Ga (MSWD=1.10) (Fig. 5C).

## DISCUSSION

### Total Pb/U age of NWA 2977

From the U–Pb isotopic analysis, the crystallization age of NWA 2977 and 773 was estimated to be around 3.1 Ga. As described above, the age of NWA 2977 has been investigated in previous studies. Ar–Ar dating method gave an age of 2.77±0.04 Ga.^10)^ Ages of 3.10±0.05 Ga and 3.29±0.11 Ga were obtained from Sm–Nd and Rb–Sr isochrons, respectively.^11)^ *In-situ* Pb–Pb analysis of baddeleyite showed an age of 3.12±0.01 Ga.^8)^ Our result of a U–Pb age of 3.13±0.10 Ga is consistent with these ages within the analytical uncertainties, except for the Ar–Ar age of 2.77±0.04 Ga. Since the closure temperature of Ar–Ar systematics is relatively lower than that of other radiogenic dating methods,^29)^ the Ar–Ar age of 2.8 Ga more likely to be affected by secondary thermal events after crystallization.

As shown in Fig. 5A, just one data point of the phosphate occurring in the host-rock of NWA 2977 plots to the right of the concordia curve, implying the effect of the impact-shock metamorphism responsible for producing the SMV. However, the shock age could not be constrained from this measurement, since the calculated planar regression does not intersect with the concordia curve in the ^238^U/^206^Pb–^207^Pb/^206^Pb plane.

There is a possibility that the U–Pb system of phosphates had been completely reset by an impact event that occurred after the igneous crystallization.^30)^ Baddeleyite is a mineral that is more resistant to thermal resetting than phosphate. When a grain size is on the order of 10^2^ μm and a cooling rate is assumed to be 10°C/Myr, its closure temperatures is above 900°C,^31,32)^ compared to 450–550°C for phosphate.^33)^ The difference in closure temperature between phosphates and baddeleyites has been taken advantage of in dating the thermal history of lunar impact breccias (*e.g.*, ref. 34). The obtained age of 3.13±0.10 Ga is consistent with, not only the Pb–Pb baddeleyite age of NWA 2977 but also the U–Pb baddeleyite age of NWA 773 clan, 3.12±0.10 Ga.^35)^ Shaulis *et al.* also reported that a U–Pb phosphate age of NWA 773 was 3.11±0.03 Ga, identical to the age of the baddeleyite. We therefore conclude that the U–Pb systematics of the phosphates in NWA 2977 were not significantly affected by secondary metamorphism.

In conclusion, the U–Pb phosphate age of NWA 2977 obtained in this study is consistent with the crystallization age of NWA 773 clan. In addition, no clear difference was found in the obtained ages of the OC and BX lithologies for NWA 773. These results indicate that various lithologies of the NWA 773 clan crystallized from a common magmatic system.^25,35–37)^

As described above, the obtained age of 3.1 Ga is relatively young compared to lunar mare basalts sampled by the Apollo and Luna missions that were in the range from 4.3 to 3.2 Ga. A previous study has suggested that the young age of the NWA 773 clan reflects more recent lunar magmatism, prolonged due to heat provided by the decay of radioactive elements such as uranium and thorium in the KREEP material.^38)^

### Cooling rate estimation after shock metamorphism

Most phosphates both in the SMV and host-rock of NWA 2977 are well fitted by a linear regression line. From the Raman spectra shown in Fig. 3, the apatite in the SMV was partially transformed into a high-pressure polymorph, tuite, indicating that the shock pressure experienced in the SMV was larger than 10 GPa.^39)^ In addition, the fine-grained matrix of the SMV crystallized from a melt of the bulk host-rock materials as described above. Based on these findings, the shock temperature recorded in the SMV is estimated to be larger than 2500 K based on the temperature-pressure phase diagram for the MgSiO_3_ system.^40)^ None of the phosphate grains in the SMV showed signs of Pb-loss, even though the rounded shape of the grains indicates marginal melting in such intense shock metamorphism as shown in Fig. 2C–E. This suggests that the cooling process following the peak shock temperature in the SMV was sufficiently fast that Pb was unable to dissipate from the grains.

In general, the mobility of elements in a grain depends not only on the maximum temperature but also on the duration of the heating and the grain size. The cooling rate of minerals (*T*′) is constrained by the following relation with closure temperature (*T_c_*), 
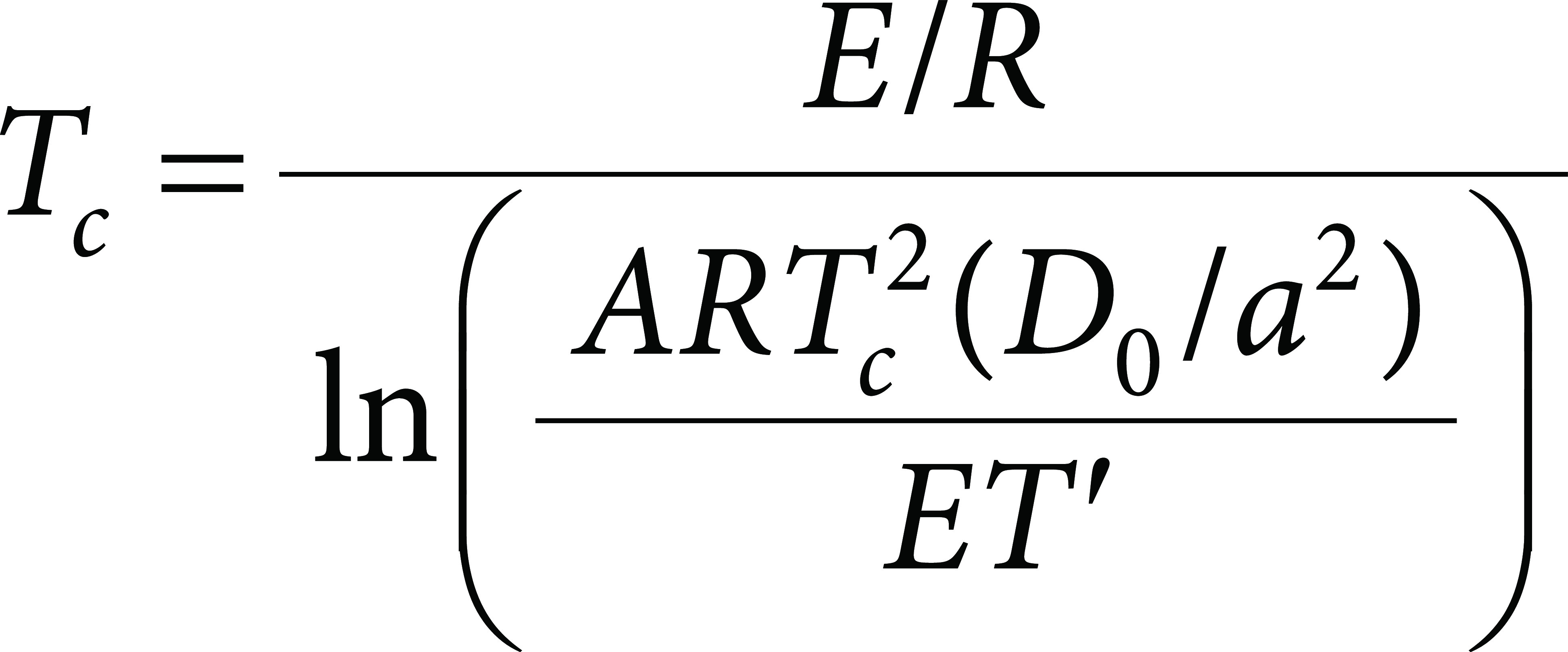
(1) where *E* is the activation energy for the diffusion process (55.3 kcal/mol), *R* is the gas constant, *A* is a constant that is dependent on geometry (for a sphere, *A*=55), *D*_0_ is a diffusion coefficient of a phosphate (2×10^−4^ cm^2^/s), and *a* is the grain radius.^32)^

If the loss of Pb did not occur at a high temperature (>2500 K), the closure temperature of the phosphate in the SMV must have been above 2500 K. Introducing *T_c_*>2500 K and *a*=5 μm into Equation (1), the cooling rate *T*′ is constrained to be larger than 140 K/s, which is consistent with a temperature decrease dominated by thermal conduction under adiabatic decompression processes.^41)^ Thus, the phosphates in the SMV must have cooled immediately after the peak shock temperature and then equilibrated with the temperature of the host-rock.

## SUMMARY

In this study, we investigated the U/Pb systematics of NWA 2977 which has a melt vein, most likely resulting from an intense shock event. Combined with the isotopic data of NWA 773 which has been considered to be paired with NWA 2977, an *in-situ* U–Pb analysis of phosphates yielded a crystallization age of 3.13±0.10 Ga. This age is consistent with the previous studies obtained from the whole-rocks and minerals of Sm–Nd, Rb–Sr systematics, and the Pb–Pb baddeleyite isochron dating. Though it is evident that the grains in the shock melt vein were exposed to intense metamorphism, the U–Pb systematics of the phosphates were not disturbed. Based on this finding, we conclude that the cooling rate of phosphate was constrained to be larger than 140 K/s.

## Notes

Mass Spectrom (Tokyo) 2023; 12(1): A0115
